# Prevalence, Genotype Distribution and Persistence of Human Papillomavirus in Oral Mucosa of Women: A Six-Year Follow-Up Study

**DOI:** 10.1371/journal.pone.0042171

**Published:** 2012-08-30

**Authors:** Jaana Rautava, Jaana Willberg, Karolina Louvanto, Lilli Wideman, Kari Syrjänen, Seija Grénman, Stina Syrjänen

**Affiliations:** 1 Department of Oral Pathology, Institute of Dentistry, and Medicity Research Laboratory, Faculty of Medicine, University of Turku, Turku, Finland; 2 Department of Oncology and Radiotherapy, Turku University Hospital, Turku, Finland; 3 Department of Obstetrics and Gynecology, Turku University Hospital, Turku, Finland; Karolinska Institutet, Sweden

## Abstract

**Background:**

Human papillomavirus (HPV) infections have been linked to a subset of oral and oropharyngeal cancers. However, little is known on the natural history of oral HPV infections. We designed the prospective Finnish HPV Family Study to assess the dynamics of HPV infections in parents and their infants. This study reports HPV genotype distribution and virus persistence in oral mucosa of the mothers.

**Materials and Methods:**

Totally, 324 pregnant women were enrolled at the 3^rd^ trimester of pregnancy and followed-up for 6 years. Oral scrapings taken with a brush were collected and HPV-genotyping was performed with nested PCR and Multimetrix® test (Progen, Heidelberg, Germany). The predictors of persistent oral HPV species 7/9 infections were analyzed using generalized estimating equation models.

**Results:**

The point prevalence of oral HPV varied from 15% to 24% during the 6-year follow-up. Altogether, 18 HPV genotypes were identified either as single or multiple-type oral infections. HPV16 was the most prevalent type at 9.7%–18.4%, followed by HPV18, HPV6, and multiple infections. Altogether, 74 women had persistent oral HPV infection determined as at least two consecutive samples positive with the same HPV genotype. HPV16 and HPV6 were the two most frequent types to persist (76% and 9%) for a mean of 18.6 and 20.2 months, respectively, followed by multiple infections (8%) for 18.3 months. An increased risk for persistent oral HPV infection with species 7/9 was associated with being seropositive for low-risk (LR)-HPV-types at baseline, whereas the use of oral contraceptives and a second pregnancy during follow-up were protective. Clinical oral lesions were detected in 17% of these women, one-third of whom had persistent oral HPV-infections.

**Conclusion:**

HPV16 and HPV6 were the most common genotypes in oral HPV-infections and were also most likely to persist. Use of oral contraceptives and a second pregnancy protected against oral HPV persistence.

## Introduction

Emerging evidence points to a causal role for human papillomavirus (HPV) in oral carcinogenesis [1], [2]. Natural history of oral HPV infections is poorly understood, and data on the HPV-genotype spectrum in the oral mucosa are scarce. Both low-risk (LR) and high-risk (HR) HPVs have been found in asymptomatic infections as well as in benign and malignant oral lesions [3].

Cross-sectional studies on asymptomatic oral HPV-infection report conflicting results on HPV-DNA prevalence, ranging from 0% to 81% with the mean of approximately 11% [4]–[11]. HPV16 seems to be the most prevalent genotype, but HPV 12, 18, 53, and 71 have also been reported [7], [8], [9], [12]. Based on a recent meta-analysis on 1,885 cases of oral cavity cancer and 2,248 oral control samples, HPV was found in 33.7% of all cancer samples, compared with only 12% of the control samples [13].

Only a few prospective studies on oral HPV-infections are available. Kurose et al. (2004) found HPV-DNA in 0.6% of oral scrapings from 662 subjects and two of them had a persistent infection over two years [12]. This is much less than recently reported in our family cohort, where 9% of the parents had persistent oral HR-HPV-infection [10]. In another recent study, 15% of 136 HIV-negative individuals had oral HPV-infections, and 60% of these infections persisted for at least six months [14]. The following risk factors of persistent oral HPV-infections have been identified: current smoking, age above 44 years, practicing oral sex, and hand warts [14]–[15].

The Finnish Family HPV Study was designed to elucidate the dynamics of oral and genital HPV- infections within families [10]. In the present report, point prevalence and persistence of oral HPV-infections are presented at the genotype level during the 6-year follow-up. The predictors of persistent species 7/9 oral HPV infections were also analyzed in univariate and multivariate models. The association of persistent infections with the development of clinical oral lesions was assessed at the study endpoint.

## Materials and Methods

### Subjects

**Figure 1:**
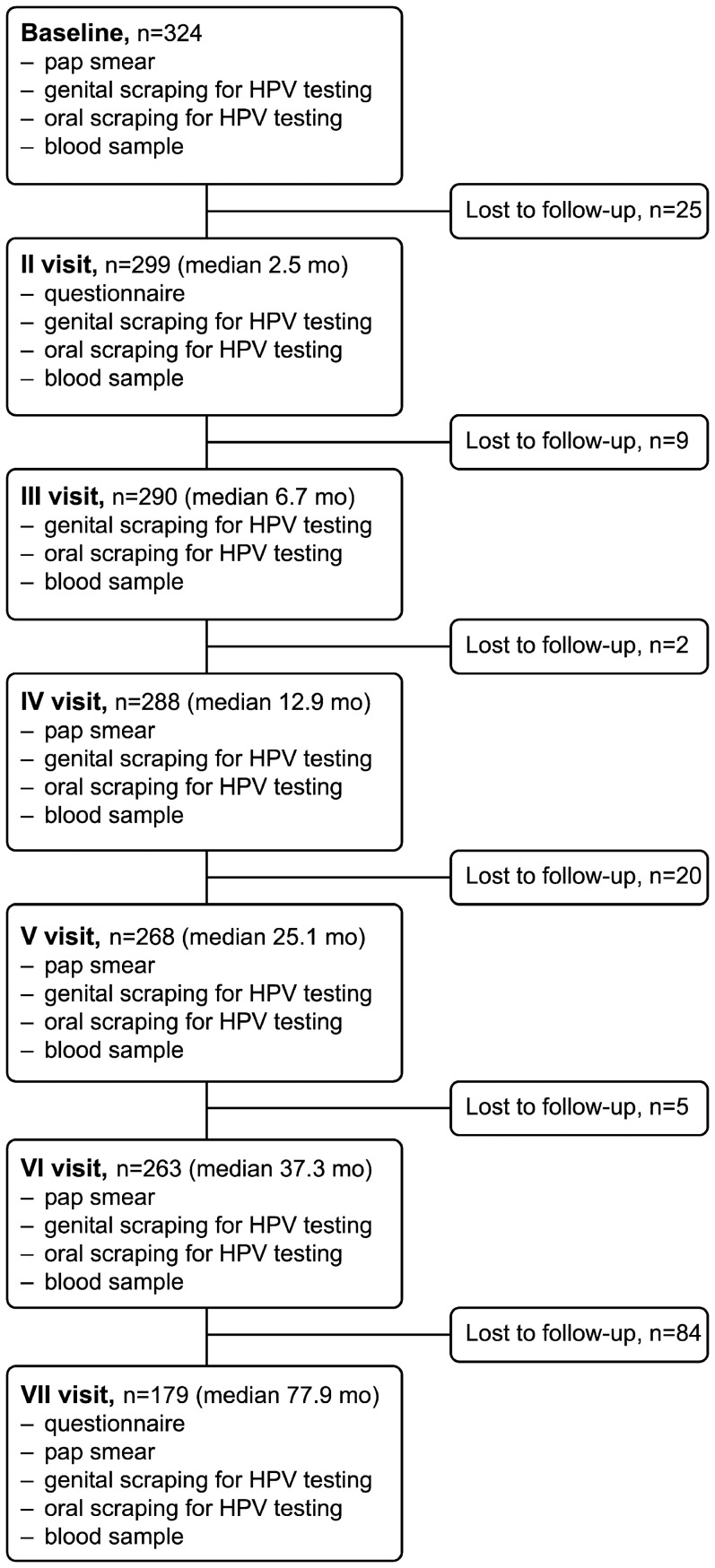
Flow chart of the Finnish Family HPV Study on oral HPV infection in women followed for six years.

The Finnish Family HPV Study is a prospective cohort study conducted at the Turku University Hospital and the University of Turku. The study protocol and its amendment (#2/1998 and #2/2006) have been approved by the Research Ethics Committee of Turku University Hospital. Altogether, members of 329 families were enrolled, comprising 329 mothers, 131 fathers and 331 newborns as described in detail previously [10]. The women were originally enrolled in the cohort at 36-weeks (minimum) of their index pregnancy and subsequently followed up (FU) for 6 years. An informed consent in written was obtained from all participants at the first visit. The present analysis is focused on oral HPV-infections among the 324 mothers who had oral swabs available. The mean age of the women was 25.5 years with a range of 18 years to 46 years (median 26.0 years). The flow chart of the present study is shown in **Figure 1**. Demographic data were collected with structured questionnaires at baseline and during FU (**Table 1**).

**Table 1:** Demographic data of the woman at baseline visit.

Characteristics	Number of women (Percentage)
**Marital status** (n = 285)	
Single	19 (6.7%)
Living with partner	132 (46.3%)
Married	131 (46.0%)
Divorced	3 (1.1%)
**Education** (n = 285)	
Compulsory school	24 (8.4%)
Vocational training	75 (26.3%)
Upper secondary school graduate	93 (32.6%)
College graduate	53 (18.6%)
Academic degree	40 (14.0%)
**Employment status** (n = 279)	
Employed	174 (62.4%)
Student	43 (15.4%)
Unemployed	62 (22.2%)
**Allergic symptoms** (n = 282)	
No	156 (55.3%)
Yes	126 (44.7%)
**Atopic symptoms** (n = 275)	
No	232 (84.4%)
Yes	43 (15.6%)
**Menarche** Mean (range) (n = 273)	12.4 (10–18)
**Abortions** Mean (range) (n = 276)	0.2 (0–3)
**Miscarriages** Mean (range) (n = 275)	0.2 (0–3)
**Deliveries** Mean (range) (n = 284)	1.3 (1–4)
**Age at first sexual intercourse** (n = 285)	
<13 years	7 (2.5%)
14–16 years	160 (56.1%)
17–19 years	105 (36.8%)
>20 years	13 (4.6%)
**Number of lifetime sexual partners** (n = 284)	
0–2	70 (24.6%)
3–5	90 (31.7%)
6–10	65 (22.9%)
>10	59 (20.8%)
**Number of lifetime sexual partners <20 years of age** (n = 285)	
0–2	122 (42.8%)
3–5	97 (34.0%)
6–10	46 (16.1%)
>10	20 (7.0%)
**Frequency of sexual intercourse per month** (n = 283)	
0–1	6 (2.1%)
2–4	86 (30.4%)
5–10	157 (55.5%)
>10	34 (12.0%)
**Practices oral sex** (n = 285)	
Never	57 (20.0%)
Occasionally	193 (67.7%)
Regularly	35 (12.3%)
**Practices anal sex** (n = 285)	
Never	232 (81.4%)
Occasionally	51 (17.9%)
Regularly	2 (0.7%)
**Age at onset of oral contraception** (n = 284)	
Never	22 (7.7%)
<13 years	3 (1.1%)
14–16 years	117 (41.2%)
17–19 years	114 (40.1%)
>20 years	28 (9.9%)
**Smoking history** (n = 284)	
Never	142 (50%)
Current or past smoker:	142 (50%)
1–10 cigarettes/day	82 (28.9%)
11–20 cigarettes/day	55 (19.4%)
>20 cigarettes/day	5 (1.8%)
**Use of snuff** (n = 259)	
Never	258 (99.6%)
One can per month	1 (0.4%)
**Use of alcohol** (n = 284)	
Never	28 (9.9%)
One dose per day	1 (0.4%)
One dose 2–3 times a week	28 (9.9%)
One dose per week	89 (31.3%)
One dose per month	138 (48.6%)
**History of sexually transmitted disease (STD)** (n = 323)	
No	261 (80.8%)
Yes:	62 (19.2%)
Chlamydia trachomatis	33 (10.2%)
Genital HSV	11 (3.4%)
Multiple STDs	18 (5.6%)
**History of genital warts** (n = 281)	
No	201 (71.5%)
Yes	80 (28.5%)
**Age at diagnosis of genital warts** (n = 78)	
<20 years	36 (46.2%)
20–24 years	31 (39.7%)
>25 years	11 (14.1%)
**Treatment of genital warts** (n = 108)	
No treatment	41 (38.0%)
Topical treatment	31 (28.7%)
Electrocautery	4 (3.7%)
Cryotherapy	3 (2.8%)
Laser therapy	12 (11.1%)
Surgery	1 (0.9%)
Several treatments	16 (14.8%)
**History of oral warts** (n = 278)	
Never	270 (97.1%)
Yes, no treatment	7 (2.5%)
Yes, surgical treatment	1 (0.3%)
**Skin warts** (n = 164)	
Hands	61 (37.2%)
Feet	64 (39.0%)
Multiple sites	39 (23.8%)

### Samples

Oral scrapings for HPV-testing were taken at baseline as well as at 2, 6, 12, 24 and 36-month and 6-year FU-visits. Oral scrapings were taken with a brush (Cytobrush®, MedScan, Malmö, Sweden) from the buccal mucosa of both cheeks as well as the upper and lower vestibular areas. The brush was immersed in 80% ethanol and then immediately frozen and stored at −80°C until use. Cervical samples were collected as described earlier [10], [16].

### HPV genotyping

HPV-DNA was extracted from the oral scrapings with the high salt method as described previously [17]. Originally HPV-testing for the presence of any HR-HPVs was performed using nested PCR with MY09/MY11 as external and GP05+/GP06+ as internal primers [18]. The PCR products were hybridized with a digoxigenin-labeled HR-HPV-oligoprobe cocktail (HPV-types 16, 18, 31, 33, 35, 39, 45, 51, 52, 54, 56 and 58) to determine whether the samples were HR-HPV-positive (+) or -negative (−) [19].

HPV genotyping was performed with a Multimetrix kit® (Multimetrix, Progen Biotechnik GmbH, Heidelberg, Germany) which detects 24 LR- and HR-HPV-genotypes as follows: LR-HPV6, 11, 42, 43, 44, and 70; and HR-HPV16, 18, 26, 31, 33, 35, 39, 45, 51, 52, 53, 56, 58, 59, 66, 68, 73 and 82. The earlier nested PCR products to detect any high risk HPVs was biotinylated by re-amplified with GP05+/bioGP06+-primers. The assay was performed in half of the volume given in the protocol in all steps except the final one. The labeled hybrids were analyzed with a Luminex LX-100 analyzer (Bio-Plex 200 System, Bio-Rad Laboratories, Hercules, USA). A median fluorescence intensity (MFI) of at least 100 beads was computed for each bead set in the sample. The cut-off value for each run and HPV-type was 1.5× background MFI (negative control)+5MFI.

If any sample was positive for HPV16, then the protocol was repeated from the original sample using nested PCR and a bead-based HPV16 genotyping assay [20]. This assay was performed to rule out possible contamination with HPV16 during the previous tests due to several amplifications and the frequency of HPV16 in different samples.

### Statistical analysis

All statistical analyses were run using the SPSS® (SPSS, Inc., Chicago, USA) and STATA (Stata Corp., College Station, TX, USA) software packages (PASW Statistics for Windows, version 18.0.1 and STATA/SE 11.0) by KS. Frequency tables were analyzed using the χ2-test with the likelihood ratio or Fisher's exact test for categorical variables. Differences in the means of continuous variables were analyzed using non-parametric (Mann-Whitney or Kruskal-Wallis) tests for two and more independent samples, respectively.

#### Outcomes of oral HPV infections and type-specific persistence

At the first level, the genotype-specific outcomes of HPV infection in each woman were assessed by comparing the viral events at each follow-up visit to the baseline HPV status. Genotype-specific persistence was denoted when any case with two or more consecutive follow-up samples were positive for the same individual HPV genotype as a single infection or as a part of multiple-type infection.

#### Predictors of type-specific persistence in a GEE model

To analyze the predictors of genotype-specific HPV persistence, we determined the predictors of persistence only for the most prevalent HR-HPV-types, i.e., those of species 7 (HPV-types 18, 39, 45, 59, 68, 70 and 85) and species 9 (HPV-types 16, 31, 33, 35, 52, 58 and 67).

A generalized estimating equation (GEE) analysis was used with panel data, clustered by women-ID and run using population-averaged (PA) model [21]–[22]. The dependent variable was binomial (persistence: yes/no), and hence, the logit link function was used. The independent within-group working correlation structure was the best-fitted covariance pattern, defined by QIC (Quasi-likelihood Information Criterion) [22]. In all models, the robust variance estimator (of 95% CI) was used to account for the within-subject correlation. In univariate GEE-models, we first tested all covariates recorded at baseline and previously implicated as potential risk factors for HPV-infections in this cohort [10]. In the final multivariate GEE-model, only the variables that were significant in the univariate model were entered, adjusted for age (continuous variable). All statistical tests performed were two-sided and declared significant at the P-value<0.05 level.

### Clinical examination

At the 6-year visit, a careful examination of the oral mucosa was performed for 179 women by two dentists (JW and LW). Mucosal changes were recorded and photographed using a digital intra-oral camera (Planmeca Oy, Helsinki, Finland). All lesions in the skin of the face and hands (e.g., warts) were also carefully inspected and photographed.

## Results

### Type-specific point prevalence

The point prevalence of oral HPV infection varied from 15% to 24% during the 6-year follow-up. Totally, 18 different HPV genotypes were identified in oral mucosa. Of these genotypes 12 HPV types were present as single infections (**Table 2**). Six additional HPV types were detected as part of the multiple type infections; i.e. HPV types 35, 39, 51, 52, 53 and 57. During the follow-up totally 48 multiple type infections were detected and HPV16 was present in 79% of them. **Table 2** presents the HPV genotype specific point prevalence at each FU-visit. At baseline, 17% (n = 55) of the oral samples tested HPV-positive. The single most frequent genotype was HPV16 at 10.5% (n = 34), followed by HPV6 at 2.2% (n = 7) and HPV66 at 0.9% (n = 3). Multiple infections comprised 1.5% (n = 5). HPV16 was present in all of these multiple infections.

**Table 2:** The genotype-specific point prevalence of oral HPV infection in women followed for 6-years.

	Baseline		2 mo		6 mo		12 mo		24 mo		36 mo		72 mo	
	N	%	N	%	N	%	N	%	N	%	N	%	N	%
HPV+ (any)	55	17.0	65	21.7	70	24.1	54	18.8	62	23.1	41	15.6	27	15.1
HPV−	269	83.0	234	78.3	220	75.9	234	81.2	206	76.9	222	84.4	152	84.9
**Genotype (% of all samples)**														
HPV6	7	2.2	0	0	2	0.7	2	0.7	1	0.4	2	0.8	0	0
HPV11	1	0.3	0	0	1	0.3	0	0	0	0	0	0	0	0
HPV16	34	10.5	55	18.4	47	16.2	28	9.7	37	13.8	32	12.2	24	13.4
HPV18	1	0.3	2	0.7	5	1.7	2	0.7	5	1.9	2	0.8	0	0
HPV31	0	0	0	0	1	0.3	0	0	0	0	0	0	0	0
HPV33	0	0	0	0	2	0.7	0	0	1	0.4	0	0	0	0
HPV45	0	0	0	0	0	0	1	0.35	0	0	0	0	0	0
HPV56	1	0.3	1	0.3	0	0	4	1.4	0	0	1	0.4	0	0
HPV58	3	0.9	1	0.3	0	0	7	2.4	0	0	0	0	0	0
HPV59	0	0	0	0	2	0.7	0	0	0	0	0	0	0	0
HPV66	3	0.9	2	0.7	0	0	0	0	3	1.1	0	0	0	0
HPV70	0	0	0	0	2	0.7	1	0.35	0	0	0	0	0	0
**Multiple HPV types (% of all samples)**	5	1.5	4	1.3	8	2.7	9	3.1	15	5.6	4	1.5	3	1.7
Distribution of HPV species, genotypes both from single and multiple type infections														
Species 5 **(HPV26,51,69,82)**					1									
Species 6 **(HPV**30**,53,56,66)**	4		6				6		4		3		1	
Species 7 **(HPV18,39,45,59,68,70,**85**)**	3		5		17		11		16		4			
Species 9 **(HPV16,31,33,35,52,58,**67**)**	44		58		58		43		55		36		28	
Species 10 **(HPV6,11,**13**,44,**55,74**)**	10				3		4		3		2		1	

The distribution of oral HPV infections according to the species are also presented.

HPV types of the species covered by the Multimetrix® test are bolded.

At the 2^nd^ visit (median: 2.5 months), the HPV-prevalence increased to 21.7% (n = 65); it increased even further by the 3^rd^ visit (6.7 months) to 24.1% (n = 70). At the 4^th^ (12.9 months) and 5^th^ (25.1 months) visits, the overall prevalence slightly decreased, but multiple infections increased from 3.1% (n = 9) to 5.6%. At the time point of 37.3 months, the overall prevalence decreased to the level of 15.6% (n = 41) with HPV16 comprising 12.2% (n = 32). After 6 years (77.9 months), HPV- prevalence was unchanged at 15.1% (n = 27), with HPV16 comprising 13.4% (n = 24) of these infections. Multiple infections decreased to the baseline level, 1.7% (n = 3). Species 9 was the most frequent HPV-species through the 6-year FU, followed by species 10 and 7.

### The duration of viral persistence

Totally, genotype specific oral HPV-persistence was found in 20 women (6.1%) when the HPV-positivity only of the baseline and last sample was regarded as selection criteria. However, during the follow-up 74 women were identified who had two or more consecutive follow-up samples positive for the same individual HPV genotype as a single or as a part of multiple infection (**Table 3**). HPV6 and 16 were the only genotypes shown to persist in more than two women with means of 20.2 months (n = 7) and 18.6 months (n = 56), respectively. Multiple infections persisted for nearly the same time: 18.3 months (n = 6, range 4.3–46.5). Infections by species 10 genotypes persisted longer than species 9 infections, at 24.6 months (n = 8, 2.4–75.8 months) and 18.4 months (n = 58, 2.1–81.2 months), respectively.

**Table 3:** The duration of the genotype and species specific persistence of oral HPV infection in women.

	N	Mean (months)	Range
**HPV6**	7	20.2	2.4–75.8
**HPV11**	1	55.2	
**HPV16**	56	18.6	2.1–81.2
**HPV33**	1	11.9	
**HPV58**	1	15.2	
**HPV66**	2	33.4	2.8–63.9
**Multiple-type infections**	6	18.3	4.3–46.5
**Species 6** (**HPV**30,**53,56,66**)	2	33.4	2.8–63.9
**Species 9** (**HPV16,31,33,35,52,58**,67)	58	18.4	2.1–81.2
**Species 10** (**HPV6,11**,13,**44**,55,74)	8	24.6	2.4–75.8

HPV types covered by the Multimetrix® test are bolded.

### Predictors of species-specific persistence

In the univariate GEE-model, being seropositive for LR-HPV at baseline increased the risk of persistence (P = 0.021; OR = 0.49, 95% CI 0.27–0.89 for seronegative women), whereas the second pregnancy during FU (P = 0.001, OR = 0.30, 95% CI 0.15–0.60) decreased the persistence (**Table 4**). In the multivariate GEE model adjusted for age, these two variables maintained their significance, and an additional predictor was disclosed: the use of oral contraceptives (P = 0.025, OR = 0.29, 95% CI 0.10–0.85), which decreased the risk of persistence.

**Table 4:** Predictors of species 7 and 9-specific persistent* oral HPV infections in GEE modeling^1^ run in a univariate mode and as adjusted for significant covariates.

Covariates	Persistent species 7/9 HPV infections					
	Crude	95%	P	@Adjusted	95%	P
	OR	CI		OR	CI	
Age	0.98	0.91–1.06	0.712	0.95	0.83–1.08	0.476
Mother seroconverted to HR-HPV (yes ref)	1.14	0.59–2.20	0.684			
Mother seroconverted to LR-HPV (yes ref)	1.42	0.77–2.61	0.260			
Mother seropositive to HR-HPV at baseline (yes ref)	0.81	0.42–1.55	0.528			
Mother seropositive to LR-HPV at baseline (yes ref)	**0.49**	**0.27–0.89**	**0.021**	**0.39**	**0.17–0.89**	**0.027**
Baseline genital HR-HPV DNA status (+ref)	1.47	0.70–3.07	0.302			
Baseline PAP smear (<ASCUS ref)	1.17	0.50–2.71	0.714			
Marital status at baseline (single ref)	0.90	0.60–1.35	0.627			
Employment status (employed ref)	1.07	0.76–1.51	0.679			
Age at onset of sexual activity (<13 yrs ref)	0.83	0.52–1.33	0.458			
No. of sexual partners until age 20 yrs (0–2 ref)	0.97	0.73–1.29	0.875			
Life-time number of sexual partners	1.02	0.79–1.31	0.859			
No. of weekly intercourse (no trend)	0.94	0.61–1.49	0.803			
No. of deliveries in all partnerships	0.75	0.46–1.20	0.238			
Practices of oral sex (yes ref)	0.78	0.42–1.42	0.418			
Practices of anal sex (regular ref)	0.65	0.39–1.08	0.103	0.51	0.21–1.26	0.148
Initiation of OC usage (<13 yrs ref)	1.04	0.66–1.64	0.840			
OC use (Y/N) (never use ref)	0.54	0.26–1.15	0.112	**0.29**	**0.10–0.85**	**0.025**
Smoking habits (never ref)	0.62	0.34–1.31	0.120			
Initiation of smoking (10–13 yrs ref)	0.81	0.45–1.48	0.509			
Consumption of alcohol (no ref)	2.96	0.97–9.13	0.059	1.44	0.25–8.31	0.678
History of STD (yes ref)	1.23	0.60–2.54	0.561			
History of genital warts (yes ref)	0.64	0.35–1.15	0.138	1.30	0.44–3.83	0.634
History of oral warts (no history ref)	1.09	0.25–4.71	0.907			
Second pregnancy during FU visit (no ref)	**0.30**	**0.15–0.60**	**0.001**	**0.42**	**0.19–0.96**	**0.042**
Change of marital status during FU	1.01	0.79–1.26	0.995			
No of current sexual partners (no trend)	1.09	0.30–3.90	0.892			

Species 7 HPV genotypes: 18, 39, 45, 59, 68, 70, and 85. Species 9 HPV genotypes: 16, 31, 33, 35, 52, 58, and 67; *Binary outcome (persistent/not persistent), as defined by persistence of the two original HPV species (same genotype) during the follow-up;

1 Results obtained from GEE with logit link for binary outcomes clustered by woman-ID, 95% CI calculated by robust estimation; @adjusted for age and all significant (and borderline) univariates in the model.

### Clinical examination

**Figure 2:**
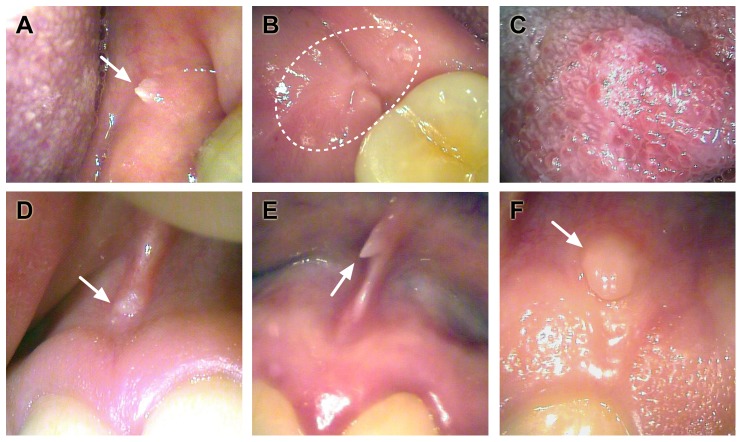
Oral mucosal changes of the women detected at the 6-year follow-up visit. **A.** A small papilloma lesion on the buccal mucosa. HPV16 and 58 were detected in the oral mucosa at the 1-year follow-up visit. **B.** Whitish hyperplastic lesion surrounded by mild erythema in the mandibular retromolar area. This woman had a persistent HPV16 infection detected at baseline and again at the 12 and 36-month and 6-year visit. She also had HPV58 at baseline and HPV56 at the 6-year visit. **C.** Hyperplastic mucosal changes in the tongue. This woman had persistent HPV16 infection at 12, 24 and 36 months. **D.** Hyperplastic maxillary labial frenulum with persistent HPV16 infection at the 24 and 36-month and 6-year visits. HPV11 was detected at baseline. **E.** A small papillomatous lesion in the maxillary labial frenulum. This woman had HPV16 at baseline that persisted at the 6-month and 6-year visits. **F.** Small hyperplastic lesions at the edge of the attached gingiva and oral mucosa. She had persistent HPV16 infection at the 12-month and 6-year visits, the latter accompanied by HPV6.

At the 6-year FU-visit (n = 181), clinical oral lesions were found from 56 (31.3%) women. The most common clinical findings were lumps (n = 8, 14%), hyperplasia (n = 7, 13%), hyperkeratosis (n = 6, 11%), and papilloma (n = 5, 9%). Oral scrapings tested HPV-positive in 28.6%, 28.6%, 100% and 40%, of these women, respectively. The results of oral HPV-testing in women with hyperplastic lesions are summarized in **Figure 2**. Taking all oral lesions together, a statistically significant association was found with smoking during pregnancy (P = 0.002, OR = 4.4, 95% CI 1.7–11.6), smoking and the use of oral contraceptives (P = 0.050), and consuming meat (P = 0.009), but there was no statistically significant association between all oral lesions and HPV-DNA-detection.

## Discussion

Persistent HR-HPV-infections are the single most important risk factor in cervical carcinogenesis and have been recently implicated in oral and oropharyngeal carcinogenesis as well [1], [13], [23]. Until now, there have been practically no data on HPV-genotype distributions or their persistence in the oral mucosa. Similarly, the risk factors for the acquisition and persistence of oral HPV- infections are completely unknown. This study is among the first to report HPV-genotype-specific infection in the oral mucosa and is the first to assess the outcome of these infections at the genotype level in a longitudinal setting with 6 years of FU. For the first time, these longitudinal HPV-data are also linked with the development of clinical lesions in the oral mucosa during this observation period.

Before delivery, 17% of the mothers-to-be had HPV-DNA in their oral samples. This prevalence is much higher than that (2.5%) previously reported among pregnant women [24] and also exceeds the average of 11–12% determined from the literature [5]–[9]. These substantial variations in HPV- detection rates can be explained by several factors, including the sampling site, sampling method and most importantly HPV-testing techniques as evident from the data presented in Table S1, which is based on three extensive reviews on the literature and 14 recent original articles. Biopsy samples from normal mucosa have resulted in higher HPV detection rate than oral rinse samples. Oral rinse is currently widely used but it is more difficult than scrapings in controlling the quality and quantity of the collected cells. The amount of cells in oral rinse varies depending on the gurgling force and the health of oral mucosa including periodontal health (e.g. grade of inflammation). Furthermore, the origin of the HPV positive cells in rinse would remain unknown. One has to remember that saliva might contain even one to 10 million microbes per one gram which easily lead to the fact that most of the DNA in the rinse sample can even be bacterial DNA, especially when the storage has not been the correct one. Also the use of several primer combinations has increased the HPV detection rate as evident from Table S1. Here we used nested PCR which was reamplified for biotinylation increasing the sensitivity of the test significantly as also found in other studies presented in Table S1. To exclude HPV 16 contamination all HPV16 positive samples were retested using the original DNA and nested PCR. The DNA was extracted with high salt method [6] and thus of good quality. Also the HPV genotyping method was sensitive (Multimetrix assay) and detected 24 different genotypes which is more than in most of the earlier studies. We also collected the samples from non-keratinized oral mucosa to ensure the yield of nucleated cells. Furthermore, the samples were stored in 80% alcohol at −80C to minimize the oral bacteria load in the sample.

In the present cohort, HPV16 was the single most frequent genotype in oral mucosa at all-time points, followed by multiple-type infections. This finding is in agreement with previous reports [5], [12], [14]. It was also evident that in the oral mucosa, the HPV-genotype spectrum was similar as encountered in the genital tract [16], [24], [25]. However, LR-HPV types were more frequent in oral samples than is usually reported in the genital tract. In this same cohort, the most frequent genotypes in the cervix were HPV16, followed by HPV6, 45, 43, 58 and 70 as reported earlier [16]. In the oral mucosa, the following genotypes were found in decreasing order of frequency: HPV16, 6, 18, 56, and 66.

Altogether, six HPV-genotypes were found to persist in 21% of these women. HPV16, followed by multiple-type infection as well as HPV6, were the most frequent genotypes to persist. According to our preliminary 2-year FU-data of this cohort, 9% of the mothers and fathers had persistent oral infection by HR-HPVs [10]. The present data indicate that extending the FU by 4 years revealed substantially more persistent infections. However, the prevalence of HPV persistence is totally depending on the definition of HPV persistence and follow-up time. D'Souza et al. (2007) reported that 60% of oral infections persisted at least 6 months (two visits) in a cohort of 136 women, which seems very high to us as compared with the present long-term longitudinal study [14]. One obvious reason for this finding is the dynamics of HPV-infections: viral clearance takes longer than 6 months, and only a minor proportion of clearance events can be detected within a 6-month FU-period [14].

Oral sex has often been implicated among the risk factors of oral HPV-infections [26]–[27]. We were unable to confirm this notion in the present study, where no association of sexual habits with persistent oral HPV-infection could be determined. Our cohort is different from many other cohorts as the women here were pregnant at baseline and had a very stable relationship with their spouses during the follow-up. Seropositivity of a woman for LR-HPV at baseline increased the risk of persistent species 7/9 oral infections. The other independent predictor in multivariate GEE was the second pregnancy during FU, which decreased the likelihood of oral persistence. It is feasible to consider that there are some immunological changes that interfere with HPV-infections during pregnancy. Similarly, a history of oral contraceptive use was associated with a decreased risk of persistence of oral HPV species 7/9 infections. Oral contraceptive use, in turn, might be a surrogate for some characteristics of common low-risk sexual behavior, e.g., a stable partnership and few partners, factors which decrease the risk of persistent HPV- infections. Circumstantial evidence to support this comes from a recent analysis of this same cohort, where predictors of type-specific persistence of cervical HPV-infections were the early onset of sexual activity (<13 yrs), early initiation of oral contraceptives, smoking, and oral sex [16]. Both oral and genital HPV-infections are responsible for the total burden of HPV-carriage in the body, and the persistence of infection at these distinct anatomical sites may be interrelated.

To elucidate the clinical relevance of these persistent oral HPV-infections, all women were subjected to clinical oral inspection at their 6-year FU. Although never reported previously, such an approach gives new insights into the potential causal role of persistent HPV-infections in the development of different oral pathologies. Interestingly, all women with hyperkeratotic changes in their oral mucosa also tested HPV-positive during the FU. During this longitudinal setting, many other clinical changes characterized by mucosal proliferation were also detected among women who tested HPV-positive in their oral scrapings. Importantly, almost all of these positive cases included the HPV16 genotype as well. This leads us to hypothesize that persistent HPV16 infection in the oral mucosa is a risk factor for different types of clinical lesions on gross inspection. However, we analyzed HPV only in the oral scrapings and not from clinical lesions. Analysis of the latter would lend additional support to the causal association of HPV with these lesions.

Taken together, the Finnish Family HPV study was designed to create foundations for a better understanding of the natural history and different outcomes of oral HPV-infections at the genotype level. The present results showed that totally 18 different HPV genotypes were found in oral mucosa and HPV could also persist in oral mucosa. Because of the wide HPV-genotype spectrum which includes many rare genotypes, a significantly larger cohort will be needed to give the study enough power to conduct the risk analysis for individual genotypes.

## Supporting Information

Table S1 Studies reporting HPV prevalence in normal oral mucosa of healthy individuals since the reviews of Syrjänen and Syrjänen 2000, Kreimer and co-workers (2010) and Syrjänen et al. 2011.
(DOCX)
