# Supplementary material for: Prevalence, Genotype Distribution and Persistence of Human Papillomavirus in Oral Mucosa of Women: A Six-Year Follow-Up Study
Source: PLoS One. 2012 Aug 30;7(8):e42171. doi: 10.1371/journal.pone.0042171 (PMC3431392; doi:10.1371/journal.pone.0042171)
Supplement: Table S1 — Studies reporting HPV prevalence in normal oral mucosa of healthy individuals since the reviews of Syrjänen and Syrjänen 2000, Kreimer and co-workers (2010) and Syrjänen et al. 2011. (DOCX) [file pone.0042171.s001.docx]

**Table S1.** Studies reporting HPV prevalence in normal oral mucosa of healthy individuals since the reviews of Syrjänen and Syrjänen 2000, Kreimer and co-workers (2010) and Syrjänen et al. 2011.

| **Reference (Country)** | **Material** | **HPV detection method** | **HPV prevalence** | **Genotypes** |
| --- | --- | --- | --- | --- |
| Syrjänen and Syrjänen, 2000, review | n=1929, oral mucosal biopsies and scrapings | Southern Blot hybridization, dot-blot hybridization, PCR. | 11% | HPV6,7,11,16,18 |
| Kreimer et al., 2010, systematic review | n=4070, oral samples cancer-free patients | PCR | 4.5% | HPV16,18,31,33,35,  39,45,51,52,56,58,59,66 |
| Syrjänen et al. 2011 | n=2248, oral biopsies or mucosal swabs | PCR (except three papers with in situ hybridization | 12% (biopsy samples 24.6%, swabs 7.2%) |  |
| Kristoffersen et al., 2012 (Norway) | n=50, buccal mucosal swabs | Nested PCR with HPV L1 gene specific primers | 56% | HPV6, HPV11, HPV16, multiple type |
| Elango et al., 2011 (India) | n=46 ”normal oral mucosa” | PCR, consensus, HPV16 primers, p16 IHC | 0 | - |
| Migaldi et al., 2012 (Italy) | n=81, oral smears | nested PCR | 1.2% | HPV90 |
| Saghravanian et al., 2011 (Iran) | n=18, ”normal oral mucosal tissue” | PCR to detect HPV16,18,31,33 | 0 | - |
| Szarka et al., 2009 (Hungary) | n=72 normal-appering oral mucosa, exfoliated cells | MY/GP PCR, genotyped by restriction analysis of amplimers | 4.2% | HPV11, HPV16 |
| Horewicz et al., 2010 (Brazil) | n=56 with periodontitis, n=26 with gingivitis, n=22 healthy periodontium | RT-PCR for HPV16 | 0 | - |
| Esquenazi et al., 2010 | n=100 oral brushes | PCR | 0 | - |
| Kreimer et al., 2011 (US, Mexico, Brasil) | n=1,688 men, oral rinse | Roche Linear Array | 4.0% | HPV16,31,35,39,52,  55,56,58,59 |
| Gillison et al., 2012 (US) | n=5,579, oral rinse | PCR and type-specific hybridization | 6.9%  (men 10.1%, women 3.6%) | 37 different genotypes, HPV16 was the most common |
| Turner et al., 2011 (US) | n=151, saliva samples | PCR for HPV16 and HPV18 | 2.6% | all HPV16 |
| Bottalico et al., 2011 (US) | n=317 older men, oral rinse | MY09/MY11, FAP59/64 primers, dot blot hydridization/direct sequencing | 37% | 46 alpha-HPV  108 beta-HPV  14 gamma-HPV |
| Ragin et al., 2011 (US) | n=118 women |  | 10.2% |  |
| Matsushita et al., 2011 (Japan) | n=196 female sex workers, oral cavity swabs | L1 gene by PCR GP5+/6+ primers Kurabo GeneSquare Microarray | 6.1% | HPV56,18,31,6,40 |
| Sánchez-Vargas et al., 2010 (Mexico) | n=46 women with CIN, buccal swab | PCR for HPV16 and 18 | 72% | HPV16 |
